# “People Associate Us with Movement so It’s an Awesome Opportunity”: Perspectives from Physiotherapists on Promoting Physical Activity, Exercise and Sport

**DOI:** 10.3390/ijerph18062963

**Published:** 2021-03-14

**Authors:** Kerry West, Kate Purcell, Abby Haynes, Jennifer Taylor, Leanne Hassett, Catherine Sherrington

**Affiliations:** 1 Institute for Musculoskeletal Health, Sydney Local Health District, Camperdown, NSW 2050, Australia; kate.purcell@sydney.edu.au (K.P.); abby.haynes@sydney.edu.au (A.H.); Jennifer.taylor1@sydney.edu.au (J.T.); leanne.hassett@sydney.edu.au (L.H.); Cathie.sherrington@sydney.edu.au (C.S.); 2 School of Public Health, Faculty of Medicine and Health, The University of Sydney, Sydney, NSW 2006, Australia; 3 Physiotherapy Department, The Children’s Hospital at Westmead, Sydney Children’s Hospitals Network, Westmead, NSW 2145, Australia; 4 Sydney School of Health Sciences, Faculty of Medicine and Health, The University of Sydney, Sydney, NSW 2006, Australia

**Keywords:** physical activity, sport, exercise, physiotherapy, physical therapy, health promotion, ageing, disability

## Abstract

Insufficient physical activity (PA) is a critical public health issue especially in the context of COVID-related deconditioning. Health professionals are well placed to promote community-based PA but there is little supporting implementation research. We aimed to explore physiotherapists’ knowledge, views, attitudes and experiences regarding the promotion of physical activity, exercise and sport within daily clinical practice in order to guide development of strategies to support implementation of PA promotion by physiotherapists, in particular those treating older people, and adults and children with a disability. We conducted interviews and focus groups with 39 physiotherapists. Two researchers coded transcripts with an iterative coding approach. Analysis returned five main themes: putting principles into practice; working with conflicting priorities; multiple client barriers; connections build confidence; and the battle for information. The physiotherapists accepted their legitimate role in PA promotion. Limited clinical and administrative time and acute treatment priorities often superseded PA promotion but the lack of updated information regarding suitable community-based PA opportunities and lack of trust in community providers were the biggest barriers. Strategies to enhance PA promotion by physiotherapists should address time and information constraints, and build partnership connections between health professionals and community-based PA providers.

## 1. Introduction

Increasing physical activity (PA) for all people at all ages and capacities is an urgent and critical goal in public health [1]. Leisure-time PA includes community-based recreation such as walking, cycling, and sport, as well as traditional structured exercise, and is a major factor in the prevention of non-communicable diseases. Lifetime patterns of PA are associated with a number of morbidity, disability, and mortality outcomes [2] including: adiposity; diabetes mellitus; stroke; cancer; cardiovascular and respiratory disease; cognitive, mental and bone health; and falls in older people [3,4]. In addition, there is significant economic burden and cost to the health care system associated with inactivity [5]. Despite these compelling arguments, globally three in four adolescents (11–17 years), and one in four adults (18+ years) are insufficiently active [6]. The prevalence of insufficient PA is even higher in women, older adults, and those with disability, raising significant questions of health inequity [7]. Insufficient PA is also greater in high-income countries. In Australia, less than half of adults and up to 80% of children and adolescents do not meet guidelines [8]. The United States [9] and United Kingdom report similar rates [10]. Moreover, the recent global pandemic of COVID-19 has contributed to a secondary ‘deconditioning pandemic’ [11] with shutdowns and community PA and sport cancelled.

To address this public health issue, the World Health Organization (WHO) launched the Global Action Plan on Physical Activity [12] (GAPPA) in 2018 to ensure member states meet and reduce physical inactivity by 15% (relative to 2016). In 2020, WHO published updated Guidelines on Physical Activity and Sedentary Behaviour [13] recommending an average of 60 min per day of moderate-vigorous PA for children (7–17 years), and a minimum of 150–300 min moderate or 75–150 vigorous PA each week for all adults, including both aerobic conditioning and strength training activity. Children and adults with disability are also included in the guidelines and are recommended to perform the same amounts of activity as their able-bodied peers if possible. In the guidelines, older adults (aged 64+ years) and older adults with disability are additionally recommended to incorporate multicomponent training with balance and strength training on three or more days per week to enhance functional capacity and prevent falls.

Health professionals have long been identified as crucial to the promotion of PA. They have the capacity to provide direct individual counselling and referral, and can contribute to broader policy and practice development [14]. However, while there is the need for PA promotion [15], there is limited evidence, beyond efficacy trials, for the implementation of PA interventions by physiotherapists, physicians, nurses and other allied health professionals [16]. Implementation studies and studies of interventions to enhance PA promotion and referral by health professionals are also needed. Studies in the UK have reported that health professionals use routine clinical contact to discuss PA but that frameworks, guidelines and interventions were not used consistently or with a long-term perspective [17,18]. Barriers and facilitators have been identified for both clinicians and patients in both the UK and Australia with regard to promotion of physical activity, including time constraints, lack of specific knowledge of PA guidelines, and lack of awareness of available community-based PA opportunities [19,20,21]. Physiotherapists are a group of health professionals who could be central to the implementation of PA promotion in the healthcare setting [22]. Physiotherapists are specialists in the domain of human movement and trained in exercise prescription and work in a wide variety of clinical settings and across all age groups. Previous studies have found that physiotherapists see promotion of PA as part of their clinical role [17,18,23]. In 2019, there were 10,192 physiotherapists practicing in New South Wales (NSW) [24] and around a third working in hospital, outpatient, community health and rehabilitation settings [25]. Therefore, physiotherapists have both an appetite for, and the workforce capacity to deliver, PA promotion within the NSW public health system. For these reasons, physiotherapists are the focus of this study. The study was conceived as physiotherapist-led health services partnership research, drawing on frontline and experiential understanding of issues most important to clinical practice in hospitals. Daily interaction with patients and colleagues within the health system make physiotherapists ideally placed to identify relevant clinical research questions and undertake research that has clear and feasible application [26,27]. Leadership by an ‘insider’ can also model an evidence-based practice culture and strengthen research participation by other physiotherapists and the credibility of research outputs and implementation plans [28,29]. Partnerships between physiotherapists and academics make use of complementary expertise to produce pragmatic but rigorous research [26].

This study is part of the Professional Referral to Physical Activity, Sport and Exercise (PROPOSE) project involving a quantitative survey and qualitative research with NSW health professionals to establish current levels of knowledge, clinical practice, perceived barriers, and information/training needs for PA promotion and community-based referral to exercise and sport. The findings from this research will inform a subsequent intervention design for future implementation-effectiveness trials.

The main objectives of this study were to: (1) identify the current practices of physiotherapists in regard to promotion of PA within daily clinical practice with a focus on referral to community-based structured PA opportunities for older clients and people of all ages with physical disabilities and (2) to obtain input from physiotherapists to develop and refine strategies to help physiotherapists improve PA promotion and referral. A secondary objective was to explore physiotherapists’ knowledge, views, and attitudes to promoting PA and making referrals to community-based activities.

## 2. Materials and Methods

### 2.1. Study Design

This study involved focus groups and interviews with physiotherapists working in public hospitals in Sydney. Focus groups were chosen as the best means of generating discussion about the research topic that would elicit a range of professional perspectives, experiences and ideas [30]. Focus groups can facilitate a collaborative dialogue between participants and researchers that explores different ways of seeing and solving practice problems [31]. Interaction between participants encourages reflection, elaboration and justification, thus “focus groups have the potential to reveal more about clinicians’ knowledge and reasons for particular patterns of thinking than could be obtained in one-to-one interviewing” [32].

A qualitative descriptive process was used because of its strength in providing clear information on how to improve practice [33]. Our theoretical perspective was informed by realist and pragmatist perspectives which assert that there is a social and material reality independent of human consciousness yet myriad ways of seeing and experiencing this reality. While we can never fully know the ‘truth’ of a complex social situation we can improve our understanding [34,35]. One way of doing this is by exploring and triangulating the perspectives of people who are closest to it.

### 2.2. Ethical Considerations

The study was approved by the Sydney Local Health District Human Research Ethics Committee (HREC RPAH zone) on 1 July 2019 (reference X19-0126) with research governance approvals at all sites. All participants gave written informed consent and were free to withdraw from the study at any time.

### 2.3. Recruitment

Recruitment was purposive, with the aim of achieving maximum variation in relevant workplace roles [36]. We aimed to include representation across different clinical settings, patient types and levels of acuity, and include physiotherapists with a range of years of experience. Participants were eligible for the study if they worked as a physiotherapist in a local public hospital treating older people and/or adults or children with a physical disability. Physiotherapy departments from Sydney Local Health District (*n* = 4) and the Physiotherapy and Rehabilitation departments of Sydney Children’s Hospitals Network (*n* = 4), plus the Sydney Local Health District Paediatric Physiotherapy community team, were invited to be involved via invitation to the manager. Eight out of nine departments agreed to participate. Invitations to participate in a focus group were then sent to individual physiotherapists who worked with the target groups (as identified by the manager).

### 2.4. Data Collection

A focus group discussion guide was developed to target concepts most relevant to the research aims (see Supplementary materials). These open-ended questions were informed by a previous survey of 100 health professionals at the same local health district and paediatric health network [manuscript in preparation]. and included questions about professional roles and practices relating to PA promotion, barriers and enablers to promoting PA, and ideas about how best to support increased promotion of PA. The guide was pilot tested with individual physiotherapists. In an iterative process, suggestions about PA promotion interventions were discussed with subsequent groups to test for relevance and applicability.

We intended to collect all data face to face but had to utilise videoconference for later focus groups due to COVID-19 related social restrictions. Thus we conducted five focus groups face-to-face and four focus groups via videoconference. On three occasions only one participant was available for a scheduled group so three individual interviews (via videoconference) were conducted. The same outline questions were used regardless of the mode of delivery.

All focus groups and interviews were conducted by the two lead researchers: KW (a hospital-based physiotherapy manager and doctoral candidate) and KP (a research physiotherapist). Both were relatively new to qualitative research but had extensive experience chairing forums and working with diverse stakeholders so they were able to draw on skills in active listening and managing group dynamics. The first focus group was observed by AH, an experienced qualitative researcher. KP kept field notes for each interview. The focus group facilitators debriefed following each session to discuss the group dynamics, identify strengths and limitations in their management of the process, and to reflect on key issues that suggested emergent themes.

KW knew several study participants as current or previous colleagues. We attempted to minimise social desirability bias in three ways: 1. KP took a more pronounced role in the focus group where there were collegial relationships, 2. Researchers emphasised their desire to understand and learn from participants, including taking account of the breadth of potential viewpoints and using prompts to encourage the expression of divergent views [32], and 3. KP was responsible for recruitment so no pressure was put on KW’s colleagues to participate.

Focus groups and interviews were conducted at participants’ workplaces and scheduled around staff availability where possible. All sessions were audio recorded, transcribed verbatim by a professional transcription service and corrected by KW or KP.

Data collection commenced in August 2019 and completed in November 2020, the project timeline having been disrupted by the pandemic. Data collection ceased when early analysis indicated that the sampling had been of sufficient breadth, and the data was of sufficient quality and depth, to address our research aims and allow transferability to other contexts [37,38]. Others have found that three to six focus groups were sufficient to identify most prevalent themes [39].

### 2.5. Data Analysis

Transcripts were analysed in NVivo 12 Plus (QSR International, Chadstone, Australia) [40] using a qualitative descriptive approach which seeks to present a rich but unadorned picture of participants’ views that stays close to the data and is of applied use to practitioners [41,42,43]. Three researchers (KW, KP and AH) developed a descriptive coding frame based on the a priori concepts targeted by the focus group questions (e.g., beliefs about PA promotion). They trialed this on two transcripts, working collaboratively to refine the coding frame and develop concordance about the scope and content of each code. This coding frame provided a ‘start list’ to which inductive codes were added to capture emergent concepts during this and the subsequent coding process [44]. All data was linked to individual profiles and site details to ensure it was known who said what. KW and KP coded the remaining transcripts independently, frequently discussing their views about code coherence and emergent themes.

Initial themes were developed by reading within and across the codes to look for conceptual patterns, and synthesising the points that were most relevant to the research questions [44]. KW and KP presented these initial themes with corroborating data at a workshop with the co-authors who sought to act as ‘critical friends’ [45] by challenging thematic explanations and interpretations of the data. Themes were further refined through this reflective process and through writing the themes narratively in this manuscript, with the lead author returning to transcripts and audio recordings to check the validity of our assertions. Throughout this process, analysis involved reviewing data against each other to identify conceptual differences/similarities and degrees of prevalence and strength [46,47].

Research rigour was strengthened via researcher triangulation and critical review of the developing themes as part of the constant comparative process [47,48]. Our reporting accords with the Consolidated criteria for reporting qualitative studies (COREQ) checklist which was submitted for review with this paper [48].

## 3. Results

A total of 39 mostly experienced physiotherapists, 67% female, participated in either an individual interview (*n* = 3) or a focus group (*n* = 9; group size range 2–9). Focus groups ranged in duration from 35 to 54 min and interviews from 19 to 37 min. As Table 1 shows, there was good representation across years of experience (and associated levels of seniority) and work settings.

This section first presents the five themes identified in our analysis: 1. Putting principles into practice, 2. Working with conflicting priorities, 3. Multiple client barriers 4. Connections build confidence, 5. The battle for information. We then describe the proposed strategies for improving the promotion of PA by physiotherapists, that were derived from participant suggestions.

Participant quotes are used to illustrate key points, in italics below. To preserve anonymity they are identified only by participant number and their work setting.

### 3.1. Putting Principles into Practice

There was strong agreement that promoting PA is part of a physiotherapist’s role. Participants understood the importance of participation in PA for health and wellbeing and felt it was a good fit with their skills in exercise prescription and human movement, welcoming opportunities to incorporate it in their practice.

*“I feel like people associate us with movement and it’s an awesome opportunity to be able to deliver that message”*

(P31, Paediatric acute)

*“It’s just part of being a physiotherapist. We help with function, and we help with mobility, and one way to facilitate that is to tell them to do those activities, not just when they’re seeing us in hospital, but outside so they don’t come back into hospital. … We might be one of the most knowledgeable [professions] about prescribing those type of things to the patients”*

(P4; Adult acute)

Most participants demonstrated an awareness of PA guidelines but appeared to lack detailed knowledge about the recommendations and made little reference to these when dealing with patients.

*“Oh goodness. I know it’s [recommended PA] a few hours. I think it’s like about two or three hours of physical activity, and then one hour of screen time, or something like that”*

(P37; Paediatric community)

*“The World Health Organization saying, what? 30 min of moderate to vigorous activity to do that at least five times a week, I think”*

(P13; Adult Rehabilitation)

There appeared to be a disconnect between role perception and current practice with factors such as clinical setting and the experience of the clinician influencing likelihood of successful PA promotion and referral. A few of the more experienced clinicians working in community and outpatient settings described a skill set they had built up over time incorporating “prompting behavior change”, motivational interviewing, engaging with families and carers and developing a knowledge base of local community activities.

*“I think the skills are developed with experience. I don’t think we teach that at Uni, and I don’t think junior physios have that when they walk straight into the job”*

(P3; Adult community)

Yet, even amongst the highest and most engaged PA promoters ongoing follow-up was felt to be important but rarely able to be offered. Often this was due to organisational or wider system structures such as overloaded schedules and pressures to discharge patients quickly.

*“Unfortunately we don’t really follow up, give them a call further down the track to see if they followed up on that. I guess, in an ideal world, that’s what we’d like to do to ensure that that’s followed through. But, because it’s so busy, and so time constrained, it’s not something that we can do easily”*

(P4, Adult acute)

There were noticeable differences in clinical practice between those working with children and those working with older adults. This was most strongly apparent in relation to sport which was strongly identified as only suitable for younger patients. The majority of paediatric physiotherapists described routinely/frequently having discussions with children and their families about organised sports as an accessible and fun way to increase PA. However, sport was rarely mentioned by physiotherapists working with adults who usually described referring to community-based exercise classes, encouraging walking and swimming and only referred to sporting opportunities if their client (usually a younger adult) was already involved in, or wishing to return to, sport.

*“[It’s about] getting people moving any way they can. So, I guess it’s going to be a little more population specific as to what you might consider appropriate... In an elderly population … I would say anything that is moving segments is general activity. Whether it’s something in the garden, or just moving off the couch, that’s something. But if you look at younger populations, usually you’re thinking sport, getting active, surfing, running, tennis, playing a sport, something like that”*

(P1; Adult musculoskeletal)

### 3.2. Working with Conflicting Priorities

Lack of clinician time was readily identified as a barrier to PA promotion, however this often seemed to be a matter of how priorities were set, both organisationally and in terms of individual practice. Priority was given to acutely unwell patients, particularly in the inpatient setting. Participants described organisational pressure to maintain a large caseload with a high throughput. As a result they were often unable to follow-up patients and rarely knew if any advice they had given was followed.

*“Sometimes, not always, it’s not your main focus especially when you’ve got high acuity patients and you’ve got a deteriorating chest. You’re going to prioritize that every day of the week over a sedentary person’s general exercise”*

(P21; Adult acute)

*“as an acute physio, to get someone to adhere to that, it’s a lot of behavioural change and a lot of one-on-one working. In our workload, we see them maybe once for half an hour, maybe twice, three times and they’re discharged”*

(P21, Adult acute)

There was also a strong focus on impairments when setting physiotherapy treatment goals. This conflicted with the desire to address more participation focused goals of physical activity occurring though sport or community based activities. Paediatric physiotherapists were more likely to mention a focus on fun and catering to the interests of the child whereas physiotherapists working with adults, and especially older people, had a strong focus on managing the impairment or mitigating risk.

*“…we have certain exercises that people kind of continue to do, and we have it targeted for them specifically in wanting to see how many repetitions and things they do. So it’s structuring that kind of aspect of what we do here at the hospital. I was thinking about those same kinds of things. You want instructor, whoever they might be now in a structured program to be able to kind of set up particular exercises, monitor them, monitor their progress, see if things are changing and progress”*

(P12; Adult Rehabilitation)

*“I think that’s [promoting PA] one of the main things I try to do. I think that’s my end goal really, for my kids is to not really need the therapy but to be participating in something that it’s more fun and interests them”*

(P33; Paediatric musculoskeletal)

### 3.3. Multiple Client Barriers

Physiotherapists identified a range of client barriers to increasing PA. These strongly influenced both promotion of general PA and the types of structured activities that might be suggested. Barriers included cost of activities, availability of transport, specific impairments and disabilities, lack of family support, poor motivation and availability of time.

*“…some of our patients, their mobility is quite poor. They, usually, are accompanied by carers or family members, especially, if you have kids that are working 9–5, and they’re like, “I, honestly, don’t have the time to bring my mother in”*

(P17; Adult rehabilitation)

*“Sometimes too it’s education... families can be almost resistant because they don’t think it [sport] will be suitable …some people, if they haven’t been exposed to it, or don’t have the education or anything, they can see it [sport] as not appropriate for a severely disabled child. Or think it’s too much for them, they’ve already got so much on their plate”*

(P31; Paediatric acute)

Many of the participants were working with patients with multiple co-morbidities and significant disabilities. In many cases physiotherapists appeared to feel defeated by these barriers, with few solutions suggested. There was also an identified gap for these more severely affected patients with very few community activities catering for people with severe and multiple disabilities.

*“I guess the biggest barrier is that, for clients like mine who have specific needs, there aren’t structured exercise classes…”*

(P3; Adult community)

### 3.4. Connections Build Confidence

To successfully refer or transition patients from the health service to community-based PA participants said that they needed to feel confident that their patient would be safe and the activity would cater for individual needs. Due to this, therapists often referred to activities that were designed for specific conditions, e.g., Parkinson’s groups.

*“You have to really have that disease as a specialty and for us to feel comfortable to refer to them. We have one exercise physiologist here who specialises in cancer, which is great”*

(P18; Adult acute?)

There appeared to be a general lack of trust in external community based exercise providers with concerns about the suitability of the activities and the risk of injury. There was also concern that activity providers would not be able to respond to changing needs or be able to monitor clients for adverse events. Many physiotherapists therefore preferred to find an exercise program within the health service run by a health professional if these were available. In many cases where referrals to community-based exercise providers were made the activity was run by somebody already known to the therapist.

*“… we probably tend to refer within the district. Because we know them, we know the people who run the programs. We know exactly what they’re doing. Whereas anything outside, our kind of health bubble, it’s like we don’t know what they do. We don’t know what the quality is, what the cost is”*

(P12; Adult rehabilitation?)

In one department a recreation officer was employed to assist with finding sport and PA opportunities for children. Physiotherapists in this department had a high level of trust in this person, frequently referred children to them and reported positive outcomes.

*“… here at the hospital [we] have a recreation officer. So for kids that need more specific modifications or different activities, we’ve been getting them involved more recently for things like race running and linking in with those specialised disability sporting events”*

(P35; Paediatric rehabilitation?)

Confidence could also work both ways with providers of activities not always comfortable to accept referrals from health professionals. There was also a perception by some physiotherapists that contacting providers may be seen as “stepping on toes” or trying to intimidate and so the likelihood of building trust was diminished. Despite this there were a few of physiotherapists who acknowledged that spending time “building the relationships” would result in more successful PA referral pathways.

*“And I think they probably know as much about us as we know about them, as well. They probably are very unfamiliar with the services that run here at the hospital, and it’s just that how do we bridge that gap?”*

(P11; Adult Rehabilitation)

### 3.5. The Battle for Information

Difficulty locating up to date and comprehensive information about community based activities was identified by most physiotherapists as an ongoing challenge. While some relied on word of mouth from colleagues a number of physiotherapy departments had developed resource folders or online resources with information about local services, gymnasiums, sports and exercise classes. There was a level of frustration with these methods as it was hard to keep information up to date and to source a wide variety of opportunities to suit every client. Activities in the community change frequently and it was difficult to keep abreast of which activities could be trusted in terms of safety and quality.

*“...when it’s more complicated or there are disabilities involved, that’s where I think I don’t have the resources and I’d like to know more, .... I’d literally be asking around me in our department, because I know there’s people that have the answers”*

(P31; Paediatric acute)

*“You can put it all together, but then within a couple months it can be outdated. So you know the effort to put it together and then keep it updated with all of what’s available in the community is a big task”*

(P8, Adult Aged Care)

Some of the departments involved in the study offered tertiary services with patients travelling from all over NSW to attend. In these cases it was not possible for the physiotherapists to find comprehensive information about local opportunities. The solution in this case was to refer to another physiotherapist in the local area who could assist with locating the appropriate classes or activities.

*“challenge is knowing what’s available to them. And I would push that back to the local therapists a lot because they know their community better”*

(P23; Paediatric musculoskeletal)

### 3.6. Implementation Strategies: Connections, Information and Time

In order to inform the development of implementation strategies we asked physiotherapists what might help them improve PA promotion and referral within their roles and services. Suggestions focused around making connections, finding information and allocation of time. Three main ideas were explored.

#### 3.6.1. An Online Information Portal:

Suggestions focused on the creation of a website or app which would be kept up to date externally and would become a one-stop shop for information about the availability of activities. Ideally the portal would have an interface where either the therapist or client could input specific search terms to find relevant opportunities. Searchable information could include tailoring of the activity for people with disability, location, accessibility, cost and transport.

*“I think it’d be great to have a database where you could... I don’t know if it’d be a flow chart or where you can click different options and whether they’re ambulant in a wheelchair, whether they’re fragile, whether they’re disproportionate, whether they can’t fall on their head and do scrums. Where you can pick different options in there and click the area, their geographical area and a whole heap of options come up ”*

(P33, Paediatric musculoskeletal)

#### 3.6.2. Forums for Connecting Physiotherapists, Community Providers and Clients:

These suggestions included a local expo where community providers of sport and exercise opportunities would have a chance to interact with the physiotherapists to share information, ask questions and create connections. In paediatrics, sport and disability providers running multi-sport or “come and try” days for children to experience options were suggested. These had been utilised by some departments in the past and were seen as particularly useful when held on hospital grounds so the physiotherapists were able to see children participating and meet the sports personnel.

*“we’ve got groups of kids to come in, and they all try a sport together. And we’ve had some really nice feedback from those groups, where they come in, feel quite empowered, and then go out and get more linked-in with their community groups”*

(P27; Paediatric rehabilitation)

#### 3.6.3. Dedicated PA Liaison Staff:

A final suggestion was to support departments with additional resourcing to create a specialised role devoted to finding suitable community opportunities and working with both physiotherapists and clients to identify and overcome barriers to PA participation. The suggested role could work directly with clients and/or offer support to physiotherapists via skills training (e.g., PA counseling skills) and resource development.

*I think even the presence of a dedicated service towards that, who could do brief follow ups. And then do the legwork of providing information. Resources in the community, proper referrals to where it may ever be is a lot more efficacious than us doing it. We’re juggling a lot of balls as acute physiotherapists a lot of the time”*

(P21, Adult acute)

**Figure 1:**
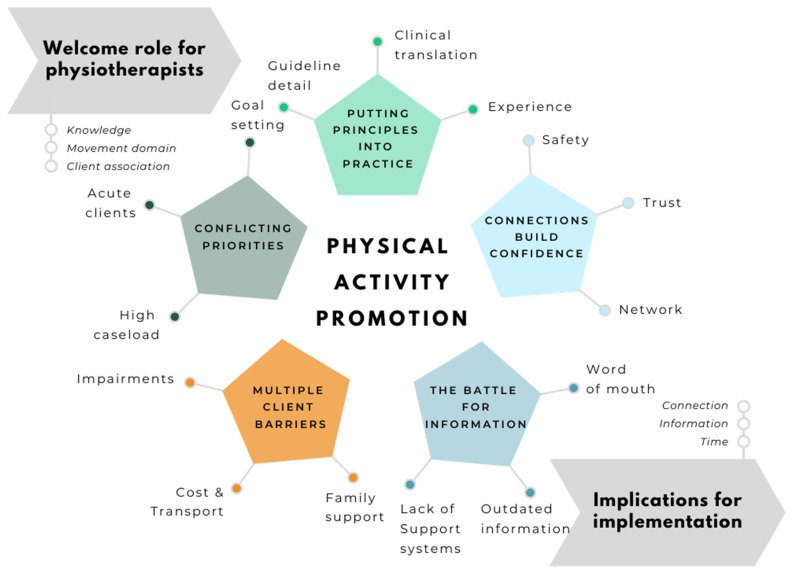
Themes for physiotherapists’ roles in physical activity promotion.

Figure 1 illustrates the major themes developed within the study, and the dimensions discussed within each theme. e. The figure depicts a flow from physiotherapist’s acknowledgement of their role in promoting PA through to suggested strategies for supporting them to realise their full potential to fulfil this role.

## 4. Discussion

The majority of the 39 physiotherapists interviewed in this study strongly agreed that physical activity (PA) promotion was in their scope of practice and that they had unique access and opportunity to promote PA with their clients. This is consistent with current literature [19,49]. Participants view themselves as specialists in the domain of human movement and trained in exercise prescription, finding PA promotion familiar, accessible, and relevant to their clinical practice. In addition, the conceptual readiness of physiotherapists matches the perceptions of healthcare clients, at least in Australia, Canada and the United Kingdom where adults report PA advice from physiotherapists is valued, expected and acceptable [22,50].

However, within the theme of *Putting principles into practice*, a gap became evident between participants’ generalised awareness of PA guidelines, and the detailed, applied knowledge and understanding required to translate the guidelines into clinical practice, particularly for client groups with acute or specialised needs. Participants acknowledged that PA promotion required skills in motivation and behavioural change but it seemed out of reach to, “…help them somehow achieve it.” Both specialisation and level of experience influenced an individual’s perception of the practicability of promoting PA. For example, physiotherapists working with children were more willing than those working with older adults to consider and include the factor of client enjoyment through sport and physical recreation. However, personal preference has been shown to be instrumental to adherence, such as the ‘f-words’ (function, family, fitness, fun, friends, future) used in childhood disability that align with the International Classification of Functioning Disability and Health (ICF) [51,52]. Two recent reviews suggest that enjoyment, social interaction and individual tailoring are important factors in PA interventions for older adults [53,54]. These factors were not identified by the majority of physiotherapists working with older adults in our study. For the physiotherapists working with older people, client safety and trust in the PA provider were paramount. Those new to their career or role appeared to either perceive PA promotion as a secondary/non-core aspect of their job and/or felt constrained by their lack of localised departmental or community knowledge. Notably, none of the participants mentioned access to existing mentoring in PA promotion, or induction/ongoing training in locally available PA programs and services.

For most participants, conflicting clinical priorities are one of the most significant barriers to PA promotion. The most commonly mentioned of these were time, client impairment, and setting of client goals. Participants experienced time constraints both in clinical contact time and non-clinical information gathering/administrative capacity. They also described a challenge common to medical triage: that the focus of their limited clinical contact time was necessarily on acuity, functional impairment or safety, according to client’s greatest need. These findings are supported by other research including in both acute and Australian settings [17,22,23,55]. This necessary medical/healthcare value system diminishes the capacity for long-term care/monitoring, and emphasises the need for non-clinical referral points and service provision, supporting our interest in improving referral away from the overloaded health system to community opportunities and programs.

As might be anticipated, there were also multiple client barriers to PA promotion and the suitability of programs/services. Restricted mobility, high dependence on others and/or service affordability were commonly cited barriers to community-based PA engagement. Participants also observed that for many of their clients, advice to attend activities could add to the existing carer burden or at least raise family concern about the client’s capacity/added stress. At least in the paediatric literature this has been confirmed with family-based research [56].

The *Connections build confidence* theme further articulated participants’ attitudes to community-based PA providers. There was some reticence to refer outside the healthcare system unless recommended by another contact within the same specialty. As with physiotherapists working with older people mentioned above, specialist units working in neurology, oncology or similar, expressed concern that programs/services would not be sufficiently tailored to individual needs or the needs of the disorder. They felt more comfortable referring a client to an exercise professional with specialised experience and that this might avoid physical pain, psychological distress and ultimately non-adherence on the part of the client. This concern is appropriate and suggests that more programs/services adequately tailored for a range of functional impairments are needed. However, this may also overlook the wide-reaching benefits of total physical activity and social participation for these client groups. Additional research is also needed to gain the views of patients and families in this regard. There is unrealised potential for partnership between healthcare and community-based PA providers. Particularly for our sub-group of people with physical disabilities the call to action for this recommendation has already been made through an earlier review [21]. A significant opportunity for public and private partnership and cross sector collaboration in implementation research also exists.

In the final theme, the *Battle for information*, it was evident that participants are not well supported by existing information resources and healthcare systems for PA promotion and referral. There is a lack of information that is current, accessible, and localised to the client. It was encouraging that recreation liaison resources were available in some areas (predominantly paediatric focused), though these were uncommon. Of note, participants with clients from rural/regional areas preferred to refer to another healthcare point rather than a relevant website, service, or information provider. This is a critical issue and key enabler. To facilitate physical activity promotion and referral, research is not only needed for interventions, but for implementation and information services to bridge the gap between healthcare and community-based physical and recreational activity providers.

This qualitative study is unique in targeting public hospital physiotherapists working with client’s with physical disability and older adults, many of whom have multiple co-morbidities. This allowed us to identify limitations faced by physiotherapists within the health system and to identify implementation strategies to increase PA promotion for these vulnerable groups. This study also adds a focus on the referral of clients to community-based PA providers to the existing literature. Another strength of the study was the comparison between physiotherapists working with children and those working with older adults with lessons to be learned for both groups. Limitations of the study include the personal connection of the lead researcher with some of the participants. Although we utilised this in our design intentionally to capitalise on clinical expertise it is possible that pre-existing relationships could result in participants being hindered in their responses. Another possible limitation was in our recruitment strategy. Although purposive, we had varying uptake to our invitation to participate. The number of physiotherapists who participated relative to those who declined was high in smaller departments, but was relatively low in the larger departments. It is possible that people who are already engaged or interested in PA promotion were more likely to participate which might have influenced our results. The number of physiotherapists and different departments was sufficient to allow us to reach data saturation, however it is a limitation of the study that all the departments, while different in size and clinical focus, were located in a metropolitan area. In addition the therapists working with adults with disabilities tended to be working with older adults. Future research could investigate physiotherapists working in smaller urban areas and rural and remote settings and those working with younger adults with disabilities such as spinal cord and acquired brain injury.

## 5. Conclusions

Physiotherapists, as well as their clients, welcome the legitimate role of health professionals in PA promotion. However, they are currently not well supported to implement this in the current healthcare environment. Implementation strategies addressing time and information constraints, and building partnership connections between health professionals and community-based PA providers are worthy of development and testing.

## Figures and Tables

**Table 1:** Participant Characteristics.

Characteristic		*n*	% of Participants
Total Participants		39	
Gender	Male	13	33
	Female	26	67
Years of Experience	0–2	4	10
	3–5	10	26
	6–10	8	21
	11–20	10	26
	>20 years	7	18
Work Setting/Clinical area *	Aged Care	3	8
	Neurology/rehabilitation	17	44
	Tertiary paediatrics	13	33
	Community	5	13
	Outpatients/musculoskeletal	9	23
	Acute inpatients	8	21
Client Age	Paediatric	17	44
	Adult	22	56

* Some participants were involved in more than 1 setting or clinical area.

## Data Availability

The data are not publicly available to maintain participant privacy.

## Supplementary Materials

The following are available online at https://www.mdpi.com/1660-4601/18/6/2963/s1, Interview Guide; COREQ Checklist.
