# Supplementary material for: “People Associate Us with Movement so It’s an Awesome Opportunity”: Perspectives from Physiotherapists on Promoting Physical Activity, Exercise and Sport
Source: Int J Environ Res Public Health. 2021 Mar 14;18(6):2963. doi: 10.3390/ijerph18062963 (PMC8000875; doi:10.3390/ijerph18062963)
Supplement: Supplementary file 1 [file ijerph-18-02963-s001.zip › Discussion Guide for Health Professional Interviews.docx]

**Discussion Guide for Health Professional Interviews**

**Introduction**

Thank you for agreeing to participate in this part of the study. This interview today is just to get some information about your thoughts and experiences in relation to the importance of promoting overall physical activity and referral of your clients to structured physical activity

The target groups we are interested in are people aged 50+ and people of any age with a physical disability. This information will assist us to develop an education intervention to assist health professional to make referrals to these opportunities.

These opportunities can include many different activities and they may be run by health professionals or by exercise leaders in the community.

Let me know at any stage if you would like me to clarify any of the questions as we go. It's likely to take around will take around 30 - 40mins. I will be recording our conversations and your identity will be kept confidential and all of the information gathered from these interviews will be anonymous. You can also have access to a transcript of this interview and add any comments.

**Focus Area One - Exploring beliefs about general physical activity**

- Can you tell me about the clients you see?
- When you hear the phrase “promoting general physical activity ” what comes to mind for you?
- Do you think facilitating physical activity is part of your role? Can you tell me why or why not? If yes, how do you approach it in your clinical practice?

**Focus Area Two- Exploring beliefs structured physical activity**

- When you hear the phrase “referral to structured physical activity” what comes to mind for you? (Prompt if needed – exercise, sport and physical recreation)
- Do you think facilitating structured physical activity is part of your role?
- Can you tell me why or why not? If yes, how do you approach it in your clinical practice?

**Focus Area Three- Key approaches, barriers and opportunities**

- What sources of information do you use to identify appropriate structured physical activity opportunities for your clients (exercise, sport and physical recreation)?
- How confident do you feel in referring clients? Can you explain your answer
- Can you tell me about the main barriers that you face? (Prompt for reasons)
- Can you think of anything that could assist you in overcoming the barriers
- Can you give me some examples of approaches that have been effective /worked well? (Prompt for reasons) Why do you think these worked well?
- We are developing some tools to assist health professionals to make referrals to structured physical activity opportunities. What do you think would be most useful?
